# Surgical Treatment of Cranial Fasciitis in an Atypical Maxillary Region of a Pediatric Patient: A Case Report

**DOI:** 10.3390/clinpract15030039

**Published:** 2025-02-20

**Authors:** Jetsi Marlen González-Fuentes, Miguel Angel Noyola-Frías, Oscar Arturo Benítez-Cárdenas, Elhi Manuel Torres-Hernández, Jessika Arlina González-Macías, Andreu Comas-García, Ricardo Martínez-Rider, Marlen Vitales-Noyola

**Affiliations:** 1 Service of Oral and Maxillofacial Surgery, Regional High Specialty Hospital “Dr. Ignacio Morones Prieto”, San Luis Potosi 78290, S.L.P., Mexico; jetmargon@gmail.com (J.M.G.-F.); manf001@uaslp.mx (M.A.N.-F.); oscar.benitez@uaslp.mx (O.A.B.-C.); elhimanuel@gmail.com (E.M.T.-H.); dra.jessika.glz@gmail.com (J.A.G.-M.); 2 Department of Oral Surgery, School of Dentistry, Autonomous University of San Luis Potosi, San Luis Potosi 78290, S.L.P., Mexico; rmrider@uaslp.mx; 3 Department of Microbiology, School of Medicine, Autonomous University of San Luis Potosi, San Luis Potosi 78216, S.L.P., Mexico; andreu.comas@uaslp.mx; 4 School of Medicine, Universidad Cuauhtémoc San Luis Potosi, San Luis Potosi 78290, S.L.P., Mexico; 5 Endodontics Postgraduate Program, School of Dentistry, Autonomous University of San Luis Potosi, San Luis Potosi 78290, S.L.P., Mexico

**Keywords:** cranial fasciitis, pediatric patient, post-traumatic, facial mass, maxillary sinus

## Abstract

**Objective:** The aim of this case report is to show the surgical treatment of cranial fasciitis in a 2-year-old patient. Cranial fasciitis is an uncommon, nonmalignant, and swiftly proliferating lesion that primarily involves the cranium, predominantly in the temporoparietal area. It mostly comprises smooth muscle tissue, connective tissue, and diverse immune cells. The lesion often manifests as an indurated, asymptomatic mass, averaging approximately 2.5 cm in size, although it may exceed 15 cm. Cranial fasciitis predominantly manifests in children below the age of 6. The diagnosis is validated via histological investigation, which identifies a benign tumor defined by the proliferation of spindle-shaped cells organized in a myxoid matrix, frequently displaying a storiform pattern. **Methods:** We present a case of cranial fasciitis in a 2-year-old pediatric patient, characterized by an atypical manifestation in the maxillary region. The lesion underwent surgical intervention resulting in total excision. **Results:** Three years after the surgery, the patient is asymptomatic and shows no signs of recurrence. Regular clinical follow-up and imaging are scheduled biannually, and the patient is anticipated to have a favorable long-term prognosis. **Conclusions:** Cranial fasciitis is a very rare benign lesion that occurs more commonly in childhood. In this case, surgical resection was effective, and three years later, the patient remains asymptomatic and free of recurrence, with a favorable long-term prognosis.

## 1. Introduction

Cranial fasciitis (CF) is an uncommon, non-neoplastic, fibroproliferative condition marked by the fast development of a solid painless lump on the scalp. The lesion generally measures 2.5 to 3 cm in size [1,2]. The predominant locations for CF include the temporal, frontal, and parietal regions, while atypical sites encompass the nasofacial, nasofrontal, and maxillary sinus areas [1,2]. In certain instances, CF may demonstrate the capacity for transcranial invasion, resulting in increasing neurological impairments due to mass effect or dural involvement, and may manifest with symptoms including exophthalmos, proptosis, and diplopia. Cranial fasciitis mostly impacts the pediatric demographic, with most instances arising between 3 weeks and 6 years of age, averaging 5.2 years. Prevalence of this disease is not fully described since there are no official published data, and in our country, there are no reports of similar cases. Nonetheless, cranial fasciitis has also been documented in adults, with a greater prevalence in males than in females [2,3]. The precise etiology of cranial fascitiis remains unidentified; however, it has been categorized into four probable origins: post-traumatic, radiation-induced, inherited (arising from spontaneous chromosomal translocations), and idiopathic [4,5,6]. Numerous studies indicate that men are more prone to developing CF post-trauma, while women exhibit a greater incidence following radiation therapy [1]. CF has been linked to traumatic cranial injuries, especially those resulting from obstetric interventions, such as forceps utilization during delivery [1,2].

The diagnosis of cranial fasciitis is validated via histological analysis, which generally demonstrates a well-defined lesion consisting of a loose proliferation of stellate and fusiform fibroblasts embedded in a myxoid matrix. Supplementary characteristics are hemorrhagic foci, sclerosis, aberrant mitotic figures, elevated cellularity, and pleomorphism. Immunohistochemical analyses can enhance the diagnosis, with markers such as HHF35, AML, and Ki-67 expressed in CF [7,8,9]. Cranial fasciitis presents radiologically as a well-circumscribed mixed lesion that neither invades nor extends into neighboring osseous structures [8]. The differential diagnoses for cranial fasciitis encompass pathologies with great diversity, such as proliferative fasciitis, proliferative myositis, fibroma, neurofibroma, fibrous histiocytoma, desmoid tumor, myxosarcoma, liposarcoma, hemangiosarcoma, rhabdomyosarcoma, nodular fasciitis, intravascular fasciitis, and cranial fasciitis of infancy, so the correct diagnosis is of utmost clinical relevance in this disease. The fast proliferation and alarming histological and cytological characteristics of these lesions might occasionally lead to the misdiagnosis of cranial fasciitis as a sarcoma [8,9].

The preferred treatment for cranial fasciitis is total surgical excision of the lesion, conducted based on the lesion’s location and size. This may entail craniectomy or curettage. Upon completion of resection, recurrence is improbable; yet, craniectomy may not represent the most effective treatment when the disease encompasses the petrous bone [8,9]. Alternative non-surgical treatments, including intralesional injections of triamcinolone acetonide, have been documented to provide complete clearance of lesions in certain instances, especially of those situated in the occipital bone. Radiotherapy and chemotherapy are typically contraindicated [8,9]. While exact statistics on the occurrence of cranial fasciitis are unavailable, recurrence rates are believed to be between 5.7% and 17.2%, generally manifesting within 6 months to 4 years post-treatment. Consequently, it is advised that all patients receive routine imaging follow-up, encompassing computed tomography (CT) and magnetic resonance imaging (MRI), to detect probable recurrence post-surgery [10]. This case report details a 2-year-old male pediatric patient diagnosed with cranial fasciitis, characterized by a lesion affecting the subcutaneous tissue of the right infraorbital and malar areas, accompanied by external cortical erosion.

This study aims to emphasize the significance of precise diagnosis and prompt treatment of this rare condition while also highlighting the atypical anatomical position of the lesion, which in this instance involved the facial mass and maxillary sinus.

## 2. Case Presentation

A 2-year-old male patient was evaluated by the Oral and Maxillofacial Surgery Service at the emergency department of Regional High Specialty Hospital “Dr. Ignacio Morones Prieto”. The patient presented with a well-defined asymptomatic swelling in the right nasolabial and infraorbital areas, which had been progressively increasing in size over the previous two weeks. The parents reported no history of fever or systemic symptoms. However, an acute episode of ocular epiphora had developed over the previous three days, raising concerns about potential involvement of the lacrimal drainage system or compression of adjacent structures.

The patient’s parents reported a history of facial trauma occurring several months prior, attributed to a fall from his own height, although they were unclear on the specific details. The physical examination revealed a localized well-defined swelling in the right upper jaw and orbital floor. Nasal airway obstruction was noted on the right side. No ulceration, spontaneous bleeding, or significant skin changes were noted in the affected nasogenic area. The assessment revealed erosion of the lateral wall of the right nasal cavity. The right eyeball showed no signs of restricted movement, and its function was normal. No clinically significant findings were observed during the intraoral examination. The patient’s family history was unremarkable, with no reported underlying medical conditions. After discussing the case and treatment options, the patient’s father provided informed consent for the proposed treatment and hospitalization.

**Figure 1:**
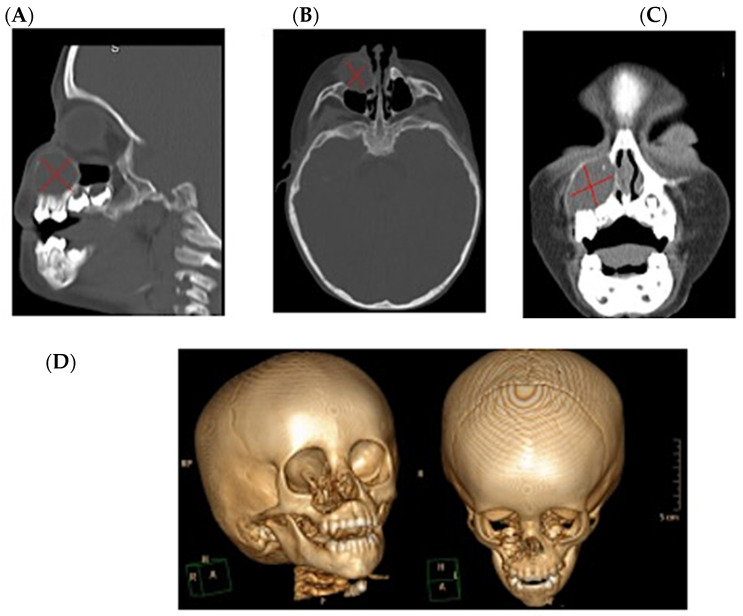
Computed axial tomography of the facial mass. Images of computed axial tomography of the facial mass of the pediatric patient; highlighted areas of the lesions are shown, lesion of 35 × 35 mm, hypodense and delimited. (**A**) In the sagittal section, erosion of the orbital floor is shown. (**B**) In the axial section, a lesion is observed in the right maxillary sinus. (**C**) In the coronal section, erosion of the lateral nasal wall is shown. (**D**) Three-dimensional reconstruction with frontal and cephalic-caudal views. X red indicate the lesion zones in tomography.

The imaging studies, including a computed tomography scan, indicated a nodular hypodense lesion on the external surface of the right maxillary ascending process. The lesion exhibited well-defined lytic borders measuring approximately 35 *×* 35 mm, leading to erosion of the external maxillary cortex and involvement of the right nasal wall and orbital floor (Figure 1). Following these findings, the patient was admitted to the pediatric service with a presumptive clinical diagnosis of a paranasal neoplasm, the specific nature of which remained undetermined. The patient subsequently underwent an initial surgical intervention under balanced general anesthesia for a biopsy, conducted through fine-needle aspiration. Before the procedure, the patient completed preoperative clinical tests to examine overall health, which included a hemogram, blood chemistry panel, general urine examination, and coagulation profile.

**Figure 2:**
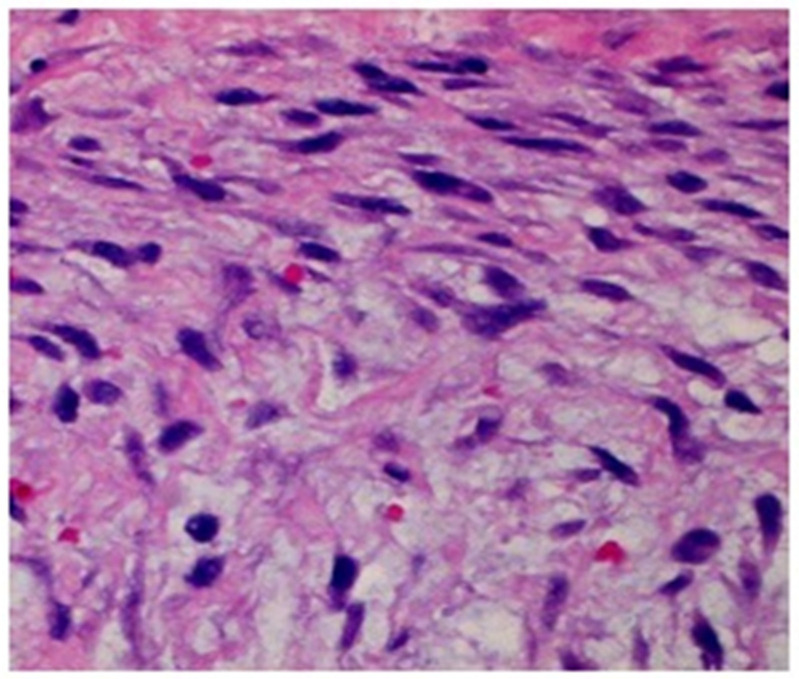
Histological analysis of the lesion. Histological sections were obtained via incisional biopsy, followed by staining with hematoxylin and eosin. A histological section reveals a myxoid matrix characterized by spindle cells, normal cellular mitosis, and the extravasation of red blood cells (100×).

The biopsy specimen was submitted to the pathology department for analysis. Histopathological analysis demonstrated tissue consisting of spindle-shaped cells exhibiting regular morphology, normal mitotic activity, and a myxoid matrix. Furthermore, a lymphocytic infiltrate and the presence of red blood cells were observed. Immunohistochemical analysis revealed positive staining for vimentin, calponin, and smooth muscle actin, confirming the diagnosis of nodular fasciitis of cranial type (Figure 2).

**Figure 3:**
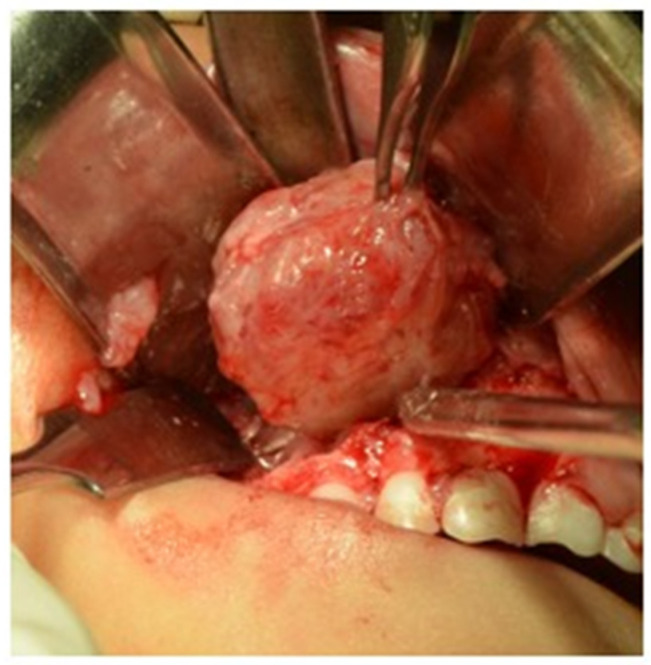
Surgical intervention for the lesion. The lesion was completely excised via blunt dissection. Surgical excision of the lesion, characterized by its well-defined and gelatinous appearance, is indicated.

Based on the histopathological results of the biopsy of fine-needle aspiration, the patient required another surgical intervention for a second biopsy, this time an excisional biopsy conducted using an intraoral circum-buccal approach on the right upper jaw. The procedure entailed careful layer-by-layer dissection to reveal and entirely excise the encapsulated lesion while minimizing disturbance to the surrounding nasal mucosa and orbital floor (Figure 3). The patient was anesthetized using balanced general anesthesia for this procedure. Although the right infraorbital nerve was close to the area to be treated surgically, it was protected during surgery, and there was no impact after surgery.

**Figure 4:**
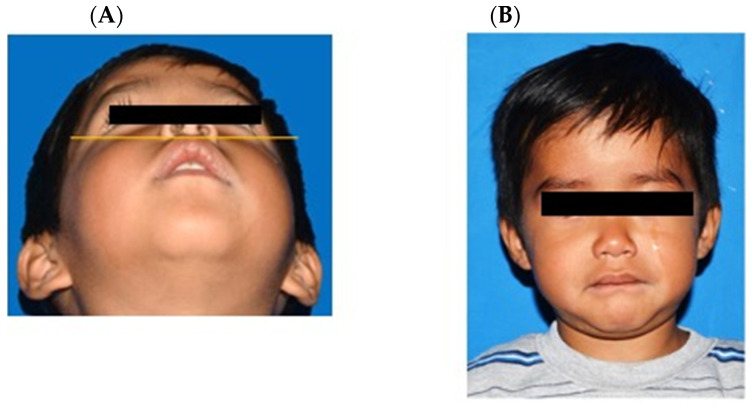
Follow-up of the patient one month after surgery. Photographs of the patient one month post-surgery. (**A**) No clinical signs of swelling are observed. (**B**) Frontal view of the patient showing no evidence of swelling.

The patient was discharged from the hospital two days post-surgery with a prescription for antibiotic therapy and analgesia, and the parents received instructions on general wound care. Seven days post-surgery, the patient was scheduled for a follow-up examination, during which a satisfactory healing process of the surgical wound was noted. The ophthalmology service evaluated the patient following hospital discharge; the patient demonstrated preserved eye movements, with no indication of reduced visual acuity. The patient has continued to undergo follow-up with regular appointments and control tomography scans over a period of three years (Figure 4). Over the course of three years, the patient has not exhibited recurrence or alterations secondary to the described lesion, indicating satisfactory progression.

## 3. Discussion

Cranial fasciitis is regarded as a variant of nodular fasciitis, exhibiting identical histological characteristics. This condition is primarily observed in the trunk and upper extremities but may also occur in the cranial region. This condition is categorized as a pseudotumor and can lead to various clinical manifestations, such as exophthalmos, diplopia, papilledema, and facial paralysis. The precise etiology of cranial fasciitis is not well-understood; however, it is thought to arise from the abnormal proliferation of myofibroblasts, frequently initiated by localized traumatic injury, though such occurrences are rarely substantiated. The condition is defined by a nodular, indurated, and generally painless lesion that exhibits aggressive growth and frequently results in the degradation of the external bone walls [11].

Cranial fasciitis predominantly occurs in children under three years, exhibiting a male-to-female ratio of 2:1; the underlying causes for this gender disparity are yet to be elucidated. The condition typically occurs spontaneously, although it may also be congenital or associated with local trauma. The subject of this case report is a male child under the age of three who sustained a fall several months prior. The parents suggested that this fall may have triggered the condition; however, they could not provide specific details as the child was actively learning to walk, and was often unsupervised [12,13].

Cranial fasciitis predominantly occurs in the head and neck region, with an incidence of approximately 7–20% of the cases. Nevertheless, erosive behavior in the facial mass is infrequently observed, with few cases documented in the maxillary–mandibular region [8,12]. The current case is noteworthy due to the presentation of a rare condition in an unusual anatomical location. The differential diagnosis must primarily include other mesenchymal lesions, including sarcomas, leiomyomas, and neurofibromas [13]. An accurate histological diagnosis is essential due to the similarity of cranial fasciitis to these conditions. Cranial fasciitis is histologically defined by a circumscribed non-encapsulated lesion featuring spindle-shaped cell proliferation within a myxoid stroma, accompanied by the extravasation of red blood cells, and immunohistochemical analysis is performed with stains for calponin and smooth muscle actin [14,15]. Therefore, histological diagnosis is essential; in our case, it was even necessary to confirm the diagnosis with a fine-needle biopsy and excisional biopsy.

Complete surgical resection is the preferred treatment for cranial fasciitis, typically resulting in a low recurrence rate of less than 2%. In our case, the patient underwent complete surgical resection, and follow-up over three years indicated a favorable outcome. Periodic imaging studies, including CT scans, have been conducted, and thus far, there is no evidence of lesion recurrence. Surgical excision achieves a cure rate of 98%, and imaging follow-up is advised to detect any potential recurrences [16]. Therefore, we consider our treatment and management of the patient to have been adequate and the surgical technique used to have been carried out in the most precise way possible so as not to damage the nearby structures and ensure the well-being of the patient, since it was a 2-year-old pediatric patient.

Due to the aggressive and erosive characteristics of cranial fasciitis, it can resemble malignant lesions. Therefore, it is essential to obtain a comprehensive clinical history and conduct a histopathological examination for a definitive diagnosis. Treatment via surgical excision is effective and generally does not necessitate additional therapies such as chemotherapy or radiotherapy [16].

## 4. Conclusions

Cranial fasciitis is a notably rare condition characterized by a low incidence rate. It should be included in the differential diagnosis of craniofacial lesions in pediatric patients with a history of facial trauma. A good diagnosis and immediate surgical intervention are essential for enhancing prognosis and improving the quality of life of pediatric patients, since it represents a surgical challenge due to patients’ young age. This case report provides valuable evidence about the positive impact of surgical interventions on children, establishing a precedent in therapeutic decisions in the area of pediatric maxillofacial surgery.

## Data Availability

The raw data supporting the conclusions of this article will be made available by the corresponding authors on request.
